# The Newcomer Health Clinic in Nova Scotia: A Beacon Clinic to Support the Health Needs of the Refugee Population

**DOI:** 10.15171/ijhpm.2018.54

**Published:** 2018-07-14

**Authors:** Graeme Kohler, Timothy Holland, Ashley Sharpe, Mandi Irwin, Tara Sampalli, Kolten MacDonell, Natalie Kidd, Lynn Edwards, Rick Gibson, Amy Legate, Ruth Ampi Kanakam

**Affiliations:** ^1^Primary Health Care, Nova Scotia Health Authority, Halifax, NS, Canada.; ^2^Newcomer Health Clinic, Primary Health Care, Nova Scotia Health Authority, Halifax, NS, Canada.

**Keywords:** Refugees, Primary Healthcare, Beacon Clinic, Care Transitions, Nova Scotia

## Abstract

Refugees tend to have greater vulnerability compared to the general population reporting greater need for physical, emotional, or dental problems compared to the general population. Despite the importance of creating strong primary care supports for these patients, it has been demonstrated that there is a significant gap in accessing primary care providers who are willing to accept the refugee population. These have resulted in bottlenecks in the transition or bridge clinics and have left patients orphaned without a primary care provider. This in turn results in higher use of emergency service and other unnecessary costs to the healthcare system. Currently there are few studies that have explored these challenges from primary care provider perspectives and very few to none from patient perspectives. A novel collaborative implementation initiative in primary healthcare (PHC) is seeking to improve primary medical care for the refugee population by creating a globally recommended transition or beacon clinic to support care needs of new arrivals and transitions to primary care providers. We discuss the innovative elements of the clinic model in this paper.

## Background

Timely access to comprehensive and continuous primary healthcare (PHC) is an essential foundation for successful integration and settlement for the refugee population.^1^ Good physical and mental health is essential for refugees to deal effectively with the challenges of resettling in a new country and participating fully in the economic, social, and cultural life in their new communities, and provides a stronger basis for refugees to adapt and thrive in their country of resettlement.^1,2^ Studies show that significantly greater proportion of refugees in Canada and internationally report physical, emotional, or dental problems than the overall population, including higher rates of TB infection, undiagnosed psychiatric problems and higher proportions of psychological illness, diabetes, maternal child health concerns, and infectious diseases.^3,4^ Lawrence and Kearns^5^ reported that challenges for the refugee population include not only access to care, but also a proper understanding of the local healthcare system and consequently the ability to navigate the system. Addressing PHC needs is considered an important aspect of the resettlement process for this population. Yet in Canada, the refugee population has experienced a persistent shortage of providers who are willing and able to accept them as patients. This has resulted in a growing number of unattached or “orphaned patients” in this refugee population.^6,7^

Responding to the arrival of the refugee population in Nova Scotia, PHC at the Nova Scotia Health Authority established a Newcomer Health Clinic (NHC) to meet the immediate healthcare needs of refugees to Nova Scotia. The NHC was established as a beacon clinic (explained in detail below) to meet the immediate healthcare needs of the population and to support transition and attachment to a primary care provider in the community.

## Beacon Clinics for Refugee Population

The Primary Care Amplification Model is an internationally recognized model of health service for refugees that aims to enhance the delivery of refugee healthcare in the PHC setting.^8^ The model identifies a central *beacon* practice with clinicians who have specific skills in delivering refugee healthcare and close ties to the community and partner organizations.^8,9^

*Beacon* clinics are outcomes-focused, providing initial health assessments for refugees with onsite interpreter services and patient education materials available in multiple languages. *Beacon* practices provide initial, transitional PHC for refugees during the first six months from acceptance, as a gateway service to full registration in the local health system.^1,10^ Patients receive a patient-owned medical record (POMR), and the *beacon* practice subsequently links patients with local providers for ongoing primary care and provides information about other health services in the community.^6^

Roles of *beacon* clinics include planning and facilitating optimal and patient-centric care in a primary care environment, sharing best practice knowledge with providers in the community, and supporting research to improve primary care for refugees.^9^ *Beacon* clinics provide information and resources about infectious diseases, immunization needs, and policies, which help to up-skill community clinics and providers in their capacity to deliver care to refugees locally. As the *beacon* clinic helps community providers and clinics in improving their confidence in offering quality care to the refugee community, it also helps build trust between the local refugee community and community practices. A typical role held by these clinics during care transition points could include: a first visit may be offered from the *beacon* staff to support successful transition into community practice; discussion of specific issues with medical and administrative staff of the accepting clinic to support better understanding of common and specific challenges related to the patient and the refugee population in general; information may also be provided to administrative staff about access to booking interpreters and other relevant resources in the community to improve experience of medical appointments and the overall ongoing care.

Since supporting transition to primary care practice or provider is a critical function of NHC, a high level review of literature was conducted to understand some of the challenges to successful transition of this population.

## Challenges Related to Transitions for Beacon Clinics

The time-sensitive health needs of refugees require an integrated community-based PHC intervention that includes support for navigating the local health system, culturally appropriate care and successful integration.^1^ To support connecting refugees with longer-term primary care, transition clinics have been established. By enabling longer visits with health professionals who are trained to work with refugee population, and providing interpreter services, refugee health clinics can eliminate barriers and challenges commonly faced when accessing health services in their resettlement communities. Transition clinics, with their settlement partners, help ensure that newcomer patients and families understand health information and the local health system, and can engage in full partnership in decision making in their healthcare.^11^ Wait times to see a health provider were shown to decrease by 30% with the introduction of a dedicated clinic.^1^ The likelihood of refugees being referred to physician specialists decreased by 45%, but those referred to specialists were shown to be the patients who were more likely to require multiple referrals due to complex medical needs.^10^

Refugees may experience challenges booking appointments, attending appointments on time, and following management and referral advice, all of which create further challenges in accessing ongoing care in community family practices.^12^ Lack of family physicians in community practices willing to accept new patients implies increased utilization of resources in the health system such as visits to a number of providers including community health centres, emergency departments, or walk-in clinics, requiring each provider to individually reconstruct health histories in the short consultation window and resulting in disjointed care and duplication of service.^4^ These issues, combined with the language barriers, time constraints, and lack of cultural competency from providers, can result in a reluctance to “share” one’s personal history for fear of the impact on a refugee claim.^4^

Given that appointment times are longer in the refugee populations, the fee-for-service system creates financial disincentives for family physicians in community practice accepting these patients into their practice.^1^ Staff in community family practices face significant challenges providing quality care for refugees when there is limited support available to inform care delivery.^13^ Re-location of refugees away from points of arrival has been associated with increased rates of temporary registration in community family practices, which can remove some financial incentives for family physicians accepting refugee patients, such as incentives for performing longer-term interventions, immunization and cervical smear tests with this population.^3^ Other barriers to follow-up in community family practices were identified by Alarcon et al,^14^ including health literacy, accessibility, transportation, lack of culturally competent providers, and issues with healthcare insurance coverage.

Lack of medical history was reported by community family physicians as problematic.^15^ Other challenges include cultural barriers, refugee population’s understanding of next of kin, and providers’ lack of familiarity and comfort working with interpretation services.^13^ Health literacy, transportation, local language proficiency, and health literacy were identified by Alarcon et al^14^ as challenges which also impact follow-up completion rates among refugee patients at community family practices. Some refugee patients also reported to feeling afraid to go to the doctor because they felt unwanted or a burden on resources.^7^ Initial health visits are often met with ambivalence on the part of refugees.

Language was discussed by refugee respondents as a significant barrier to care, particularly in situations where there was no interpreter, such as phoning a practice to arrange an appointment.^16^ Over half of health providers interviewed by Tamblyn et al^17^ reported that they perceived that language barriers led to refugees utilizing health services only when they are very sick.

## The Nova Scotia Beacon Clinic Model for Refugee Population

Consistent with trends across Canada and as a service redesign effort to meet the needs of the refugee population in Nova Scotia, PHC at the Nova Scotia Health Authority established an NHC in 2015. The NHC was implemented to serve as a beacon clinic to conduct health assessments and bridge the immediate primary care and priority needs of the newcomer population. Since its inception, improving access and care experiences for patients of the clinic has been a focus and a priority for PHC. Consequently, PHC and Public Health at the Nova Scotia Health Authority, the Immigrant Settlement Association of Nova Scotia (ISANS), the Halifax Refugee Clinic and local family physicians collaborated with the mutual goal of providing integrated, comprehensive, culturally and language appropriate primary and preventative health services for refugees in the Halifax area.

The NHC team is made up of family physicians, registered nurses and administrative support. This team is focused on providing health assessment and comprehensive primary care services for refugee population. These services are available for government assisted refugee, privately sponsored and claimants.

## Optimizing the Role of NHC as a Beacon Clinic in Nova Scotia

Since its establishment, NHC has assumed and implemented several of the core roles of a beacon clinic and these include: Primary and priority medical care, supportive clinics, care transitions, enabling provincial supports, providing advocacy support and improving academics and evidence. Many of the challenges and considerations discussed in the literature have become important design elements of the NHC model as shown in Figure and described in this section.

**Figure:**
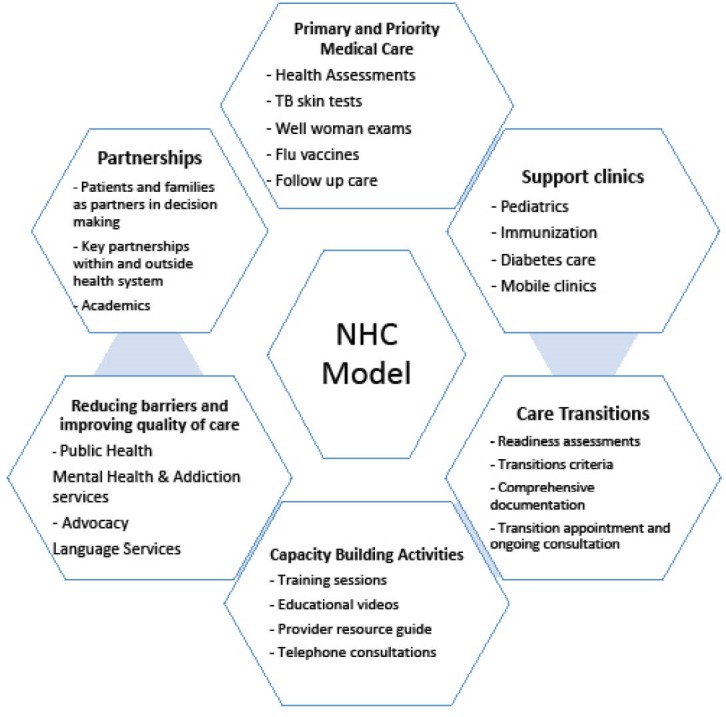
Newcomer Clinic Model. Abbreviation: NHC, Newcomer Health Clinic.

### i. Primary and Priority Medical Care

The NHC provides comprehensive primary medical care services including urgent needs, chronic disease management, preventive care and other priority services. This practice is also attuned to the health needs of this population and has expertise among its providers to deliver screening for infectious disease, awareness of communicable disease endemic to different countries, and awareness and familiarity with the clinical guidelines for immigrants and refugee population.^18^

### ii. Supportive Clinics

In addition to local expertise at the clinic, NHC has made an effort to organize and coordinate with community partners and providers to organize more comprehensive services for its patient population. Through a variety of part-time clinics, patients of the NHC in Halifax are able to access culturally appropriate services in their primary language. These include immunization clinics, well women clinics, and pediatric clinics. These three are part of regular access to shared care, whereas mobile clinics have been established when need arises. Mobile clinics offer primary care access in temporary locations and, to date, have been limited in use to when high numbers of new arrivals are expected in a short period of time.

### iii. Transitions

The model of care for the NHC is that patients remain in the care of NHC as per the transitions criteria that has been has been developed by the clinic and is currently being tested in a formal research study. The overall objective is to ensure the transitions process meets the needs of the patients and follows the comfort and readiness level of patients to engage in this process. Patients are informed of the NHC process of transition when they first arrive in the clinic and actively participate in a transition assessment that determines their ability to be transitioned to a primary care practice in the community.

### iv. Capacity Building Activities

Recognizing the importance of care transitions for its patients, the NHC team has taken a strong role in supporting capacity building activities for community providers and provincial partners including primary care providers through the development of educational videos, a provider resource guide as well as telephone consultation access to NHC team.

### v. Reducing Barriers and Improving Quality of Care

The NHC advocates within the health system and with partners outside the system to address gaps in care. This can include discussions with federal counterparts as well as developing partnerships to address local issues such as access to dentistry. These needs will shift over time, but remain as a core tenant of the work of this team.

The NHC has access to in-person interpretation, telephone interpretation and online video interpretation. The majority of visits are attempted to be scheduled with in-person interpretation as body language and other cultural nuances are best recognized through this manner. When in-person interpretation is unavailable, telephone interpretation may be accessed based on patient or provider preference. Both services are available 24/7 in many different languages. Working with interpreters is a skill learned over time and the providers in the NHC make every effort to share this skill with their colleagues across the health system.

NHC continues to identify and build partnerships with priority care and services for its patients such as improving access to mental health and other services.

The NHC is committed to providing placements for students with a range of health disciplines including nursing and medical students, and Family Practice residents.

### vi. Partnerships to Improve Quality of Care for Patients

There are many partners who have enabled the success of the NHC over the past 3 years since its inception. The Immigrant Services Association of Nova Scotia and the Halifax Refugee Clinic are two key partners who work outside of the formal health system. Both have played key roles in guiding and informing priority directions for the clinic. Similarly, Public Health represents a partnership internal to the health system that has provided training and ongoing immunization support among other aspects of importance to this patient population.

Another unique partnership developed by this team is working with patients and families to inform program planning and service delivery. A quality team for the clinic has been established with representation from clinic staff as well as patients of the clinic as equal partners in decision making processes.

While NHC continues to establish and optimize its role as a beacon clinic, based on an early and informal engagement of patients of the clinic, improving clinic processes related to care transitions was identified as a priority. A formal research study is currently in place to support the establishment of readiness, transitions and post transition role for the clinic.

## Discussion and Conclusion

The time-sensitive health needs of the refugee population requires an integrated community-based PHC approach that includes support for immediate and priority primary medical care needs following settlement, navigation of the local health system, culturally appropriate care and a process to support the permanent attachment to a primary care provider.^1,19^ To enable appropriate, patient-centric and relevant care, transitional or beacon clinics have been established across Canada and internationally. Research has shown that beacon clinics can have a significant positive impact in meeting priority assessment and care needs of this vulnerable population. Research has also shown that many of these beacon clinics are facing bottlenecks leading to increased wait times and lack of necessary supports to attaching to a primary care practice or provider. In this paper we have discussed the implementation of a beacon clinic, namely the NHC in Nova Scotia.

The NHC is an innovative model implemented to address the needs of the refugee population in Nova Scotia. Since its inception, efforts have been underway to better understand and enhance the quality of care and priority needs for patients of the clinic. Specifically, the NHC has tried to tackle some of the identified challenges in the literature and from experiences in other beacon clinics in Canada. Towards these objectives, the clinic staff have made every effort to seek input from patients, families and relevant stakeholders such as community groups and community providers to better meet the needs and identified priorities for their patients. As an example, an area of priority identified by patients, care team and PHC is the attachment to a primary care provider and a primary care clinic. An early area of focus for NHC has thus been reviewing and finding ways to effectively transition NHC patients to a primary care provider. NHC has assumed a role in the care of patients that goes well beyond offering priority medical care for patients. These activities include education and training of clinic staff and community practices in dealing with mental health needs, physician to physician communication about transitioning patients (clinic physician to community practice physician), supporting the first appointment preparedness activities for the receiving clinics such as arranging interpreters to be present, a reminder call made by newcomer clinic to confirm first appointment. Another example is the creation of a quality team that includes patients and family advisors from the clinic as equal partners in program planning and decision-makers.^20^

A formal research study is currently in progress to examine the impact of the NHC’s patient centric approach to improving quality of care and meeting priority areas of preference to its patient population.

## Ethical issues

Not applicable.

## Competing interests

Authors declare that they have no competing interests.

## Authors’ contributions

GK, TH, AS, MI, LE, GR: design, implementation and evaluation of NHC model and clinic, and contributions towards the manuscript write up. MI, KM, AL, NK, RAK: NHC clinic care and operations and contributions to the write up of the manuscript. TS: Evaluation of the model and write up of the manuscript.

## Authors’ affiliations

^1^Primary Health Care, Nova Scotia Health Authority, Halifax, NS, Canada. ^2^Newcomer Health Clinic, Primary Health Care, Nova Scotia Health Authority, Halifax, NS, Canada.
